# Induction chemoimmunotherapy may improve outcomes of chemoradiotherapy in patients with unresectable stage III NSCLC

**DOI:** 10.3389/fimmu.2023.1289207

**Published:** 2023-11-27

**Authors:** Song Guan, Shufeng Zhang, Kai Ren, Xingyue Li, Xue Li, Lujun Zhao

**Affiliations:** ^1^ Department of Radiation Oncology, Tianjin Medical University Cancer Institute and Hospital, Tianjin, China; ^2^ National Clinical Research Center for Cancer, Tianjin Medical University Cancer Institute and Hospital, Tianjin, China; ^3^ Key Laboratory of Cancer Prevention and Therapy, Tianjin, China; ^4^ Tianjin’s Clinical Research Center for Cancer, Tianjin, China

**Keywords:** chemoradiotherapy, induction chemotherapy, induction chemoimmunotherapy, non-small cell lung cancer, prognosis

## Abstract

**Background:**

Currently, the value of induction chemoimmunotherapy before chemoradiotherapy (CRT) in unresectable stage III non-small cell lung cancer (NSCLC) has not been explored. This study was designed to explore the efficacy and safety of induction chemoimmunotherapy in patients with unresectable stage III NSCLC.

**Methods:**

Unresectable stage III NSCLC patients who received CRT with or without induction chemoimmunotherapy between August 2014 and December 2021 were retrospectively enrolled. Progression-free survival (PFS) and overall survival (OS) were assessed from the initiation of treatment and estimated by the Kaplan-Meier method. The potential factors affecting PFS and OS were analyzed by univariate and multivariate Cox regression models. One-to-one propensity score matching (PSM) was used to further minimize confounding.

**Results:**

A total of 279 consecutive patients were enrolled, with 53 (19.0%) receiving induction chemoimmunotherapy followed by CRT (I-CRT group), and the remaining 226 (81.0%) receiving CRT alone (CRT group). After PSM, the median PFS was 24.8 months in the I-CRT group vs. 13.3 months in the CRT group (P=0.035). The median OS was not reached (NR) vs. 36.6 months ((P=0.142). The incidence of treatment-related adverse events (TRAEs) was similar in both groups, except that the incidence of hematological toxicity was higher in the I-CRT group (77.1% vs. 58.3%, P=0.049). Compared to induction chemotherapy, induction chemoimmunotherapy demonstrated a superior objective response rate (60.4% vs. 22.2%, P<0.001) and further prolonged PFS (median NR vs. 13.2 months, P=0.009) and OS (median NR vs. 25.9 months, P=0.106) without increasing the incidence of TRAEs in patients receiving concurrent chemoradiotherapy.

**Conclusion:**

Induction chemoimmunotherapy is safe and may improve outcomes of CRT in patients with unresectable stage III NSCLC. Moreover, induction chemoimmunotherapy may further improve treatment response and survival outcomes compared to induction chemotherapy before cCRT.

## 1 Introduction

Based on the practice-changing result of the PACIFIC trial, concurrent chemoradiotherapy (cCRT) followed by consolidation immunotherapy has become the standard of care for patients with unresectable stage III non-small cell lung cancer (NSCLC) (1). However, the optimal sequence of immunotherapy remains unclear. In the surgical setting, immunotherapy can benefit patients whether used preoperatively or postoperatively (2–4), raising the question of whether upfront chemoimmunotherapy before chemoradiotherapy (CRT) could benefit patients with unresectable stage III NSCLC. However, there is a paucity of data on induction chemoimmunotherapy followed by CRT. Although a recent retrospective study demonstrated the feasibility of induction chemoimmunotherapy with target volume reduction, it was a single-arm study (5). Furthermore, given that induction chemotherapy in the pre-immunotherapy era did not further improve survival in stage III NSCLC patients receiving cCRT (6, 7), it remains unclear whether adding immunotherapy to induction chemotherapy could further improve survival. Herein, to investigate the value of induction chemoimmunotherapy before CRT and to determine whether the addition of immunotherapy to induction treatment could improve treatment efficacy, we performed this retrospective study.

## 2 Materials and methods

### 2.1 Patient selection

Patients with unresectable stage III NSCLC who received CRT with or without induction chemoimmunotherapy at Tianjin Cancer Hospital between August 2014 and December 2021 were enrolled. The inclusion criteria were as follows: 1) age ≥ 18; 2) histologically or cytologically proven stage III NSCLC; and 3) receiving CRT with or without induction PD-1/PD-L1 inhibitor plus chemotherapy. The exclusion criteria were as follows: 1) history of any cancer-specific treatment; 2) treatment with targeted therapy; 3) receipt of induction immunotherapy alone; and 4) immunotherapy concurrent with and/or after radiotherapy.

Patients were categorized into two treatment groups based on whether they received induction chemoimmunotherapy before CRT: the induction chemoimmunotherapy followed by CRT (I-CRT) group and the CRT alone (CRT) group. In the exploratory analysis, patients who received cCRT were classified into two groups according to the induction treatment: the induction chemotherapy followed by cCRT (C-cCRT) group and the induction chemoimmunotherapy followed by cCRT (I-cCRT) group. Patients’ baseline characteristics were extracted from their medical records. Individual NSCLC cases’ histological type and stage were determined according to the WHO criteria (8) and the International Association for the Study of Lung Cancer classification (8th edition) (9), respectively.

### 2.2 Drug treatment

Immune checkpoint inhibitors (ICIs) used in patients receiving immunotherapy included camrelizumab, nivolumab, pembrolizumab, sintilimab and tislelizumab. These five kinds of ICI agents have been approved for the treatment of NSCLC based on promising outcomes in NSCLC patients (10). Each patient’s chemotherapy regimen was determined by the histological type of the tumor, the clinical condition of the patient, etc.

### 2.3 Study outcomes

The primary endpoints in this study were progression-free survival (PFS) and overall survival (OS). Following induction treatment, objective response rates (ORRs) and disease control rates (DCRs) were assessed. PFS was estimated from the start of treatment to the date of the first documented event of disease progression, death without progression, or last follow-up. OS was calculated from the initiation of treatment until death or last follow-up. According to RECIST v1.1, ORR was defined as partial response (PR) plus complete response (CR), while DCR was defined as PR and CR plus stable disease (SD). Individual patients’ treatment-related adverse events (TRAEs) were evaluated according to CTCAE version 5.0. Patients underwent follow-up visits every 3 months for 2 years and every 6 months thereafter, including clinical evaluation, CT or PET, and additional investigations when clinically indicated.

### 2.4 Statistical analysis

One-to-one propensity score matching (PSM) with baseline characteristics was used to minimize confounding. Patient characteristics between treatment groups were analyzed using the Chi-square test or Fisher’s exact test for categorical variables. Survival outcomes were estimated by Kaplan-Meier analysis and evaluated by the log-rank test. The hazard ratios (HRs) and the corresponding 95% confidence intervals (CIs) were calculated with the Cox proportional hazards model. When the univariate Cox analysis yielded a P value of ≤ 0.15, the variable was incorporated into the multivariate Cox regression analysis. Subgroup analyses (age [< 65 years or ≥ 65 years], sex [male or female], WHO histology type [squamous, non-squamous, or not otherwise specified], cancer stage [IIIA, IIIB, or IIIC], chemoradiotherapy modality [sequential or concurrent], radiotherapy dose [< 54 Gy or ≥ 54 Gy], smoking history [never, former or current], ECOG performance status [0, 1 or 2]) for PFS and OS were performed to assess the consistency of treatment effects in patient subgroups. Subgroup analyses used an unstratified Cox proportional hazards model with treatment as a covariate. A P value inferior to 0.05 was considered statistically significant. All statistical analyses were performed using SPSS version 25.0 (IBM, Armonk, NY, USA).

## 3 Results

### 3.1 Baseline characteristics

**Figure 1:**
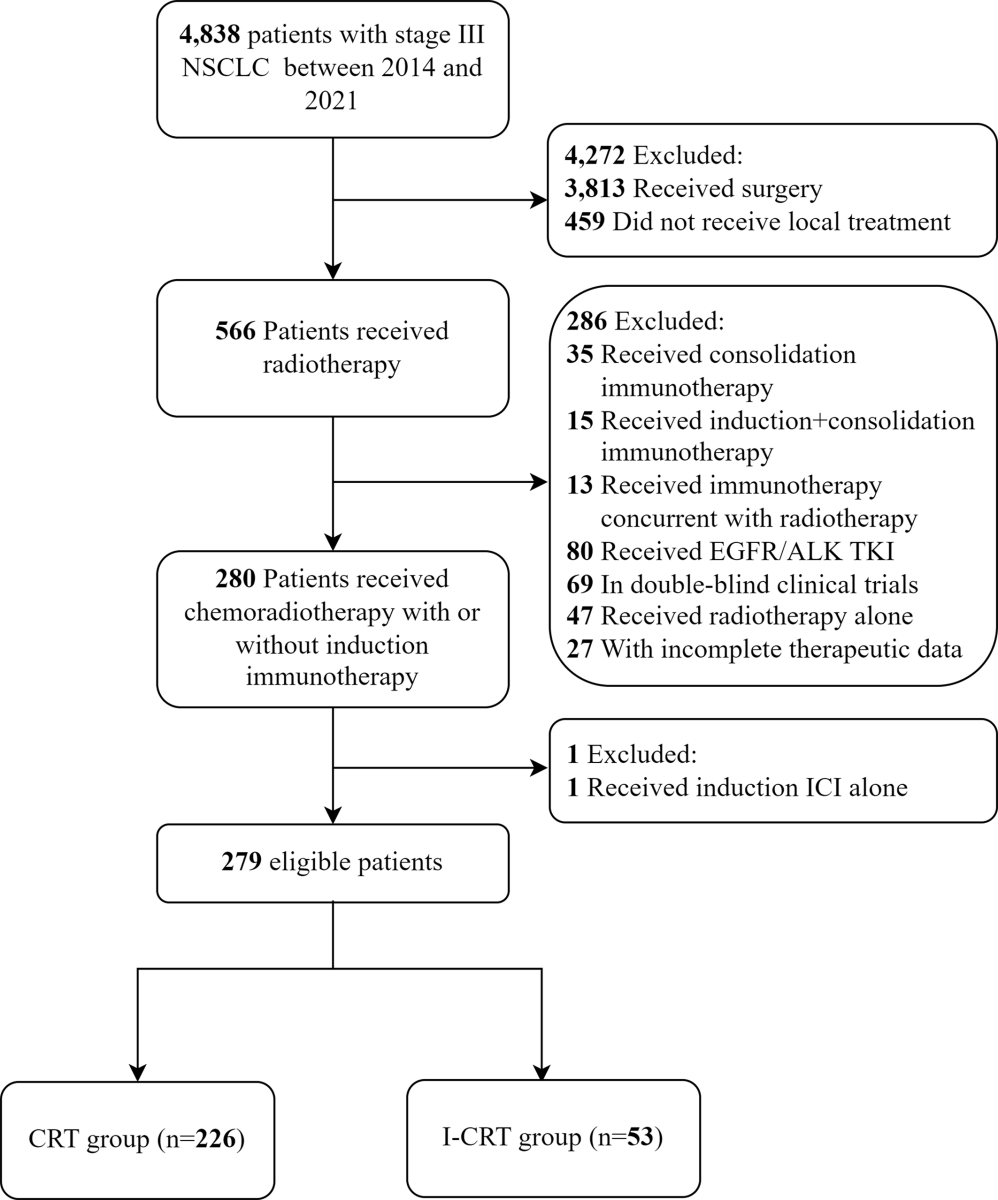
Patient inclusion flow chart.

A total of 279 consecutive patients were enrolled in this study (**Figure 1**). Among them, 53 (19.0%) patients received induction chemoimmunotherapy followed by CRT, and the remaining 226 (81.0%) received CRT alone. None of these patients received consolidation immunotherapy. Patients had a median age of 61 years and were predominantly male with an ECOG PS score of 1. Only two patients with adenocarcinoma who received induction chemoimmunotherapy had unknown driver-gene status, while the rest had wild-type driver-genes. After 1:1 PSM, the patients’ characteristics were well balanced (**Table 1**).

**Table 1:** Baseline demographic and clinical characteristics of the patients.

Characteristics	Before PSM		P	After PSM		P
	CRT *(n*=226)	I-CRT (*n*=53)		CRT *(n*=48)	I-CRT (*n*=48)	
	No. (%)	No. (%)		No. (%)	No. (%)	
Age						
<65	155(68.6)	32(60.4)	0.253	29(60.4)	28(58.3)	0.835
≥65	71(31.4)	21(39.6)		19(39.6)	20(41.7)	
Sex						
Male	185(81.9)	45(84.9)	0.600	36(75.0)	40(83.3)	0.315
Female	41(18.1)	8(15.1)		12(25.0)	8(16.7)	
WHO histology						
Squamous	150(66.4)	38(71.7)	0.261	34(70.8)	35(72.9)	0.191
Non-squamous	70(31.0)	12(22.6)		14(29.2)	10(20.8)	
NOS	6(2.7)	3(5.7)		0(0.0)	3(6.3)	
Stage						
IIIA	96(42.5)	24(45.3)	0.924	26(54.2)	22(45.8)	0.733
IIIB	109(48.2)	24(45.3)		19(39.6)	22(45.8)	
IIIC	21(9.3)	5(9.4)		3(6.3)	4(8.3)	
CRT modality						
Sequential	139(61.5)	36(67.9)	0.384	30(62.5)	31(64.6)	0.832
Concurrent	87(38.5)	17(32.1)		18(37.5)	17(35.4)	
Dose						
<54 Gy	3(1.3)	3(5.7)	0.152	1(2.1)	1(2.1)	1.000
≥54 Gy	223(98.7)	50(94.3)		47(97.9)	47(97.9)	
Smoking						
Never	38(16.8)	12(22.6)	0.319	12(25.0)	10(20.8)	0.627
Former/Current	188(83.2)	41(77.4)		36(75.0)	38(79.2)	
ECOG						
0	21(9.3)	4(7.5)	0.575	4(8.3)	4(8.3)	0.763
1	198(87.6)	46(86.8)		40(83.3)	42(87.5)	
2	7(3.1)	3(5.7)		4(8.3)	2(4.2)	

### 3.2 Treatment

All patients in the I-CRT group received induction chemoimmunotherapy, with a median of 4 cycles of induction immunotherapy (range 1-9) and chemotherapy (range 2-8). The ICI agents used included camrelizumab (15.1%, n=8), nivolumab (5.7%, n=3), pembrolizumab (20.8%, n=11), sintilimab (47.2%, n=25), and tislelizumab (11.3%, n=6). Twenty-three patients in the CRT group received CRT alone without induction chemotherapy, while the remaining 203 patients received a median of 4 cycles of induction chemotherapy (range 1-7).

### 3.3 Efficacy

In the whole population, the median follow-up from the initiation of treatment was 24.9 months (range 4.3-86.5). The median PFS and OS were 13.4 and 34.3 months, respectively. During the investigation, 213 patients developed progressive disease (PD), including 21 (39.6%) in the I-CRT group and 192 (85.0%) in the CRT group. A total of 149 patients had died when analyzed, including 8 (15.1%) and 141 (62.4%) cases in the two groups, respectively.

**Figure 2:**
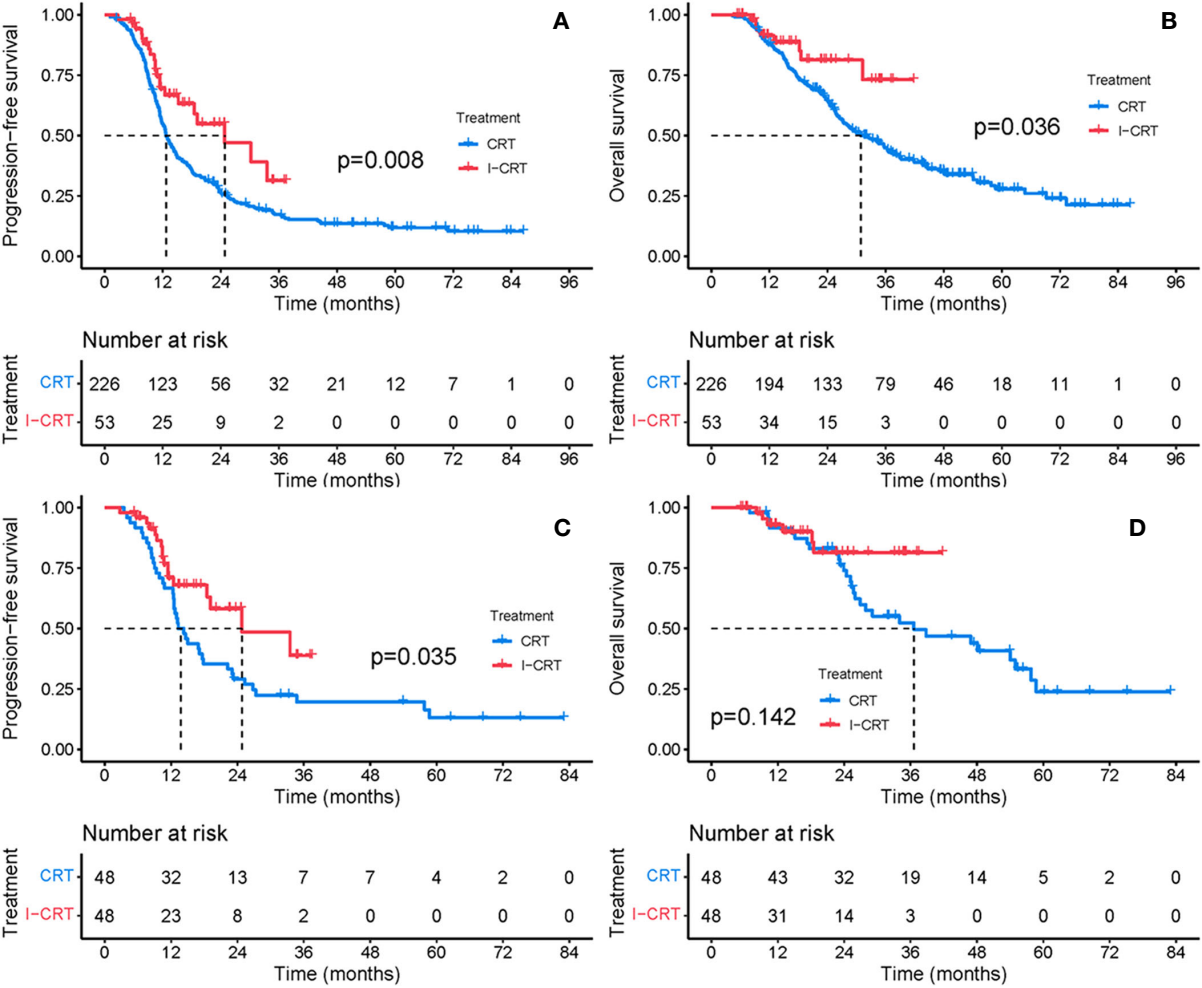
PFS and OS between the CRT group and the I-CRT group before and after PSM. **(A)** PFS from the initiation of treatment before PSM. **(B)** OS from the initiation of treatment before PSM. **(C)** PFS from the initiation of treatment after PSM. **(D)** OS from the initiation of treatment after PSM.

**Figure 3:**
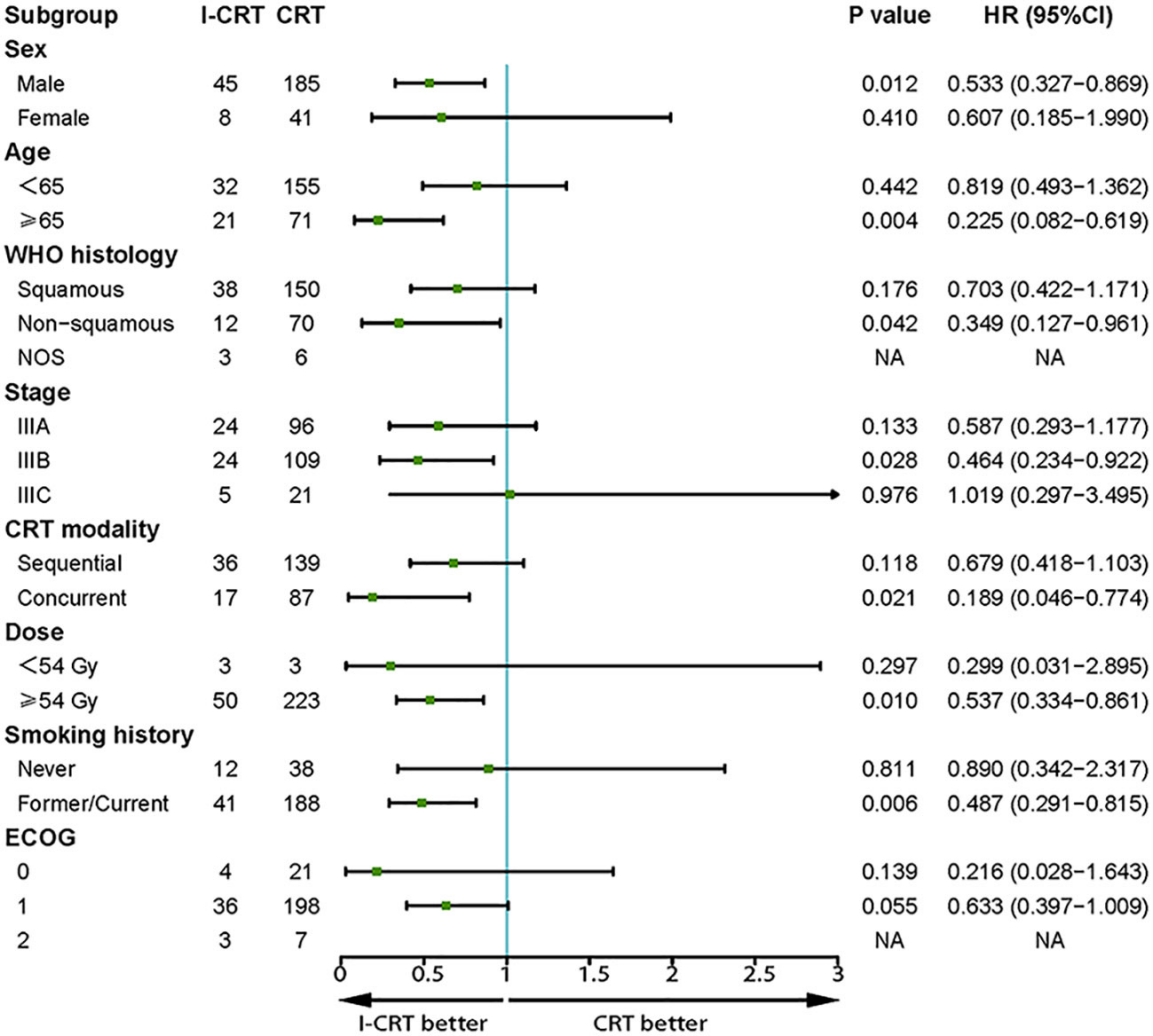
Subgroup analysis of prognostic factors for PFS in the whole population.

**Figure 4:**
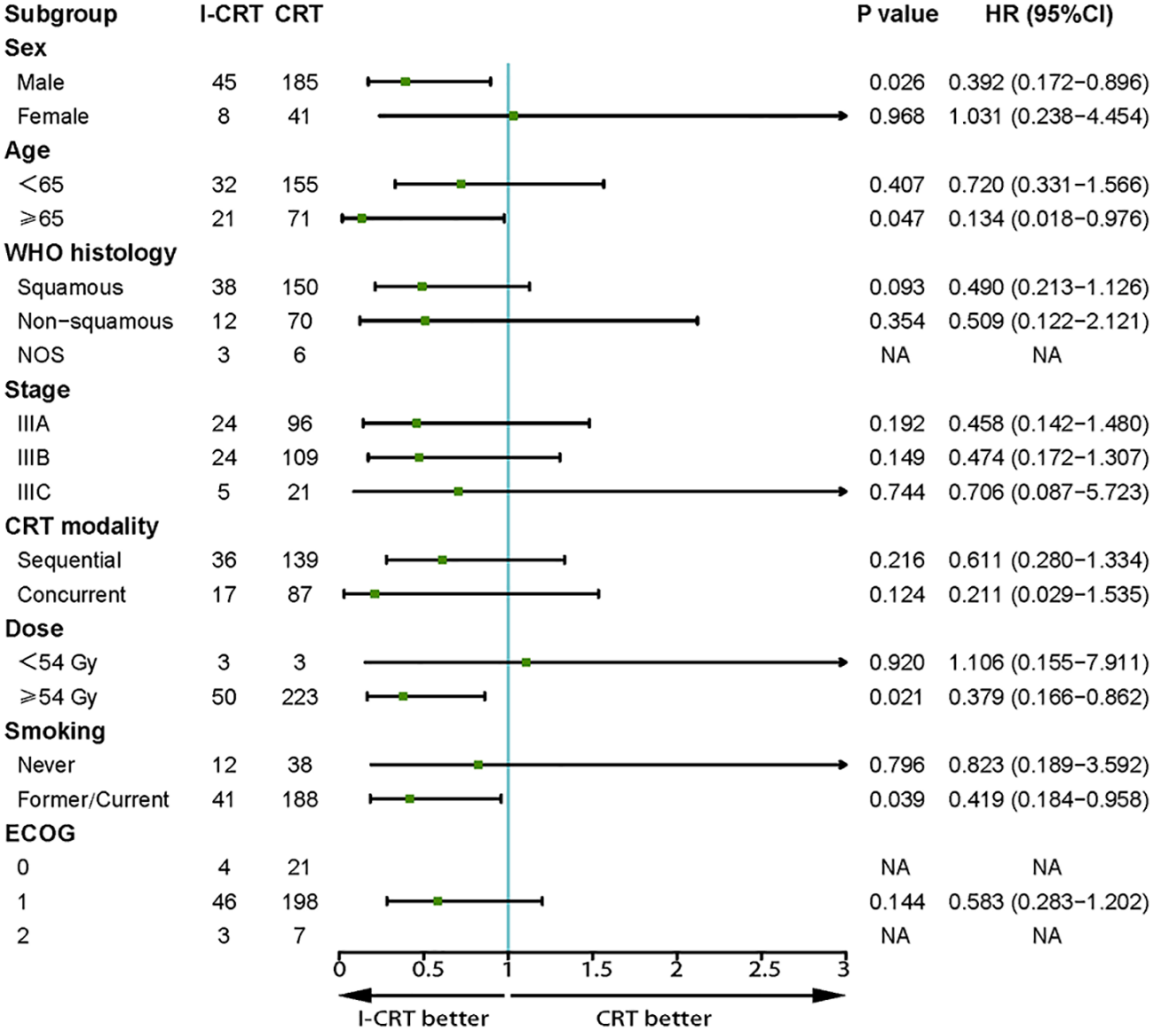
Subgroup analysis of prognostic factors for OS in the whole population.

The median follow-up for the I-CRT and CRT groups was 16.1 (range 5.3-41.8) months and 26.7 (range 4.3-86.5), respectively. The median PFS was 24.8 months in the I-CRT group vs. 12.7 months in the CRT group, with a 1-year PFS rate of 69.5% vs. 54.6% and a 2-year PFS rate of 54.8% vs. 26.6% (P=0.008, **Figure 2A**). The median OS was not reached (NR) vs. 30.9 months, with a 1-year OS rate of 91.4% vs. 88.0% and a 2-year OS rate of 81.3% vs. 64.5% (P=0.036, **Figure 2B**). The univariate and multivariate analyses further confirmed the positive effect of induction chemoimmunotherapy on improving PFS (HR=0.562, P=0.013, **Supplementary Table 1** in the **Supplementary Material**) and OS (HR=0.517, P=0.074, **Supplementary Table 2** in the **Supplementary Material**). PFS and OS benefits with induction chemoimmunotherapy were observed across most prespecified subgroups (**Figures 3**, **4**).

After 1:1 PSM, patients receiving induction chemoimmunotherapy demonstrated a consistent improvement in PFS and OS. The median PFS was 24.8 months in the I-CRT group vs. 13.3 months in the CRT group, with a 1-year PFS rate of 70.9% vs. 66.7% and a 2-year PFS rate of 58.1% vs. 29.2% (P=0.035, **Figure 2C**). The median OS was NR vs. 36.6 months, with a 1-year OS rate of 93.0% vs. 91.5% and a 2-year OS rate of 81.4% vs. 74.0% (P=0.142, **Figure 2D**).

### 3.4 Treatment-related adverse events

**Table 2** demonstrates that in the matched population, the incidence of treatment-related adverse events (TRAEs) was similar between the two groups, except that the incidence of hematological toxicity was higher in the I-CRT group (77.1% vs. 58.3%, P=0.049). In addition, one patient developed grade 1 capillary hyperplasia and 2 patients each developed dermatitis and peripheral neurotoxicity in the I-CRT group.

**Table 2:** TRAEs between the CRT and I-CRT groups.

TRAE	CRT		I-CRT		P
	No.	%	No.	%	
Whole population					
Pneumonitis	133	58.8	34	64.2	0.479
G3/4 pneumonitis	22	9.7	2	3.8	0.262
Esophagitis	42	18.6	7	13.2	0.355
G3/4 esophagitis	0	0.0	0	0.0	not applicable
Hematologic toxicity	156	69.0	40	75.5	0.356
G3/4 hematologic toxicity	55	24.3	12	22.6	0.795
Matched population					
Pneumonitis	25	52.1	31	64.6	0.214
G3/4 pneumonitis	4	8.3	1	2.1	0.358
Esophagitis	10	20.8	6	12.5	0.273
G3/4 esophagitis	0	0.0	0	0.0	not applicable
Hematologic toxicity	28	58.3	37	77.1	**0.049**
G3/4 hematologic toxicity	5	10.4	11	22.9	0.100

Statistically significant P value was written in bold font.

### 3.5 Induction chemoimmunotherapy vs. induction chemotherapy

In all patients who received induction treatment before CRT, the ORR after induction treatment was significantly higher in the I-CRT group than in the CRT group (60.4% vs. 22.2%, P<0.001), while the DCR after induction treatment was only numerically higher in the I-CRT group than in the CRT group (98.1% vs. 93.1%, P=0.292).

**Figure 5:**
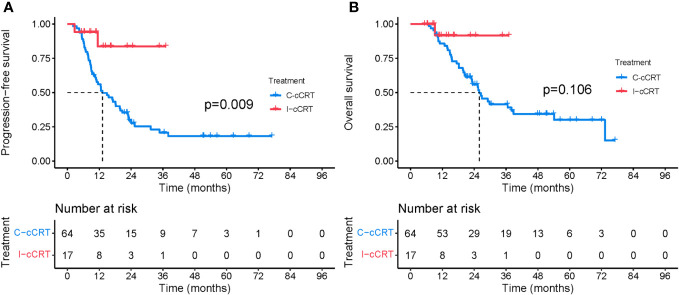
PFS and OS between the C-cCRT group and the I-cCRT group. **(A)** PFS from the initiation of treatment. **(B)** OS from the initiation of treatment.

When patients were restricted to receiving induction treatment before cCRT, i.e. induction chemoimmunotherapy followed by cCRT (I-cCRT) vs. induction chemotherapy followed by cCRT (C-cCRT), the results did not appreciably change. Baseline demographic and clinical characteristics between the two treatment groups were basically balanced (**Table 3**). The median follow-up for the I-cCRT and C-cCRT groups was 11.5 (range 5.3-37.1) and 23.1 (range 6.9-77.1) months, respectively. As shown in **Figure 5**, PFS was significantly superior in the I-cCRT group compare to the C-cCRT group (median NR vs. 13.2 months, P=0.009). One- and 2-year PFS rates were 83.7% vs. 56.1% and 83.7% vs. 29.7%, respectively. OS was numerically prolonged in the I-cCRT group than in the C-cCRT group (median NR vs. 25.9 months, P=0.106). One- and 2-year OS rates were 91.7% vs. 85.7% and 91.7% vs. 55.9%, respectively. The incidence of TRAEs was similar in both groups, except that the incidence of grade 3/4 hematological toxicity appeared to be higher in the I-cCRT group than in the C-cCRT group (41.2% vs. 23.4%, P=0.248, **Table 4**).

**Table 3:** Baseline characteristics between the I-cCRT and C-cCRT groups.

Characteristic	C-cCRT (*n*=64)		I-cCRT (*n*=17)		P
	No.	%	No.	%	
Age					
<65	51	79.7	12	70.6	0.636
≥65	13	20.3	5	29.4	
Sex					
Male	55	85.9	14	82.4	1.000
Female	9	14.1	3	17.6	
WHO histology					
Squamous	39	60.9	11	64.7	0.208
Non-squamous	23	35.9	4	23.5	
NOS	2	3.1	2	11.8	
Stage					
IIIA	25	39.1	10	58.8	0.226
IIIB	33	51.6	5	29.4	
IIIC	6	9.4	2	11.8	
Dose					
<54 Gy	0	0.0	0	0.0	not applicable
≥54 Gy	64	100.0	17	0.0	
Smoking					
Never	13	20.3	5	29.4	0.636
Former/Current	51	79.7	12	70.6	
ECOG					
0	8	12.5	1	5.9	0.389
1	55	85.9	15	88.2	
2	1	1.6	1	5.9	

**Table 4:** TRAEs between the C-cCRT and I-cCRT groups.

TRAE	C-cCRT		I-cCRT		P
	No.	%	No.	%	
Pneumonitis	38	59.4	10	58.8	0.967
G3/4 pneumonitis	7	10.9	0	0.0	0.347
Esophagitis	15	23.4	2	11.8	0.474
G3/4 esophagitis	0	0.0	0	0.0	not applicable
Hematologic toxicity	46	71.9	14	82.4	0.572
G3/4 hematologic toxicity	15	23.4	7	41.2	0.248

## 4 Discussion

Although consolidation immunotherapy after cCRT is the current standard of care for patients with unresectable stage III NSCLC, a considerable number of patients are unsuitable for or refuse to consolidation immunotherapy, resulting in the majority of patients still receiving CRT alone. In addition, not only is the proportion of patients receiving consolidation immunotherapy poor, but the proportion of patients receiving cCRT is also poor due to excessive target volumes or poor tolerability (11, 12). Despite the benefit of consolidation immunotherapy after sequential CRT, it is less than after cCRT (13, 14). Therefore, it is important to optimize the combination of CRT and immunotherapy to benefit more patients. In the surgical setting, both preoperative and postoperative application of immunotherapy can benefit patients with resectable NSCLC, raising the question of whether upfront immunotherapy before CRT could benefit patients with unresectable NSCLC. To our knowledge, this is the first real-world study to evaluate the efficacy and safety of induction chemoimmunotherapy and to demonstrate a survival benefit in patients with unresectable stage III NSCLC.

Considering that a significant proportion of patients in the real world are unsuitable for cCRT due to the high tumor burden or higher risk of pulmonary toxicity and the poor proportion of patients receiving subsequent consolidation immunotherapy, upfront immunotherapy before CRT is increasingly recommended. Compared to consolidation immunotherapy, induction immunotherapy has the advantage of shrinking the target volume to meet normal tissue constraints that allow subsequent cCRT, early treatment of distant micrometastatic disease and screening of immunotherapy-sensitive populations (15, 16). Moreover, administering a limited number of cycles of immunotherapy before CRT could greatly improve patient compliance compared to 1 or even 2 years of consolidation immunotherapy. At our center, induction chemoimmunotherapy is being attempted in a proportion of patients with a high tumor burden or a strong desire for surgery when the lesion is unresectable, providing a unique opportunity to investigate the role of induction chemoimmunotherapy. Although every eligible patient was advised to receive consolidation immunotherapy, a considerable number of patients refused because of the financial burden, fear of adverse events or the prospect of noncompliance due to 1 or even 2 years of treatment. Given the small number of patients receiving consolidation immunotherapy, and to minimize confounding by consolidation immunotherapy, we therefore excluded patients receiving consolidation immunotherapy to evaluate the efficacy and safety of the novel treatment modality of induction chemoimmunotherapy alone. Nevertheless, induction chemoimmunotherapy alone still showed promising outcomes. The addition of only a median of 4 cycles of induction chemoimmunotherapy doubled the median PFS and 2-year PFS rates in unresectable stage III NSCLC patients receiving CRT and provided a significant improvement in OS as well. Moreover, survival benefits or trends toward prolonged survival were consistently observed across most subgroups; a larger sample size could possibly have turned the trend observed in our analysis into statistical significance.

Further analysis demonstrated the superiority of induction chemoimmunotherapy over induction chemotherapy. The ORR of 60.4% for induction chemoimmunotherapy in the present study, which was similar to the 76.1% reported by Wang et al. (5), was significantly higher than the ORR of 22.2% for induction chemotherapy in the present study and the ORR of approximately 30% for induction chemotherapy in previous studies (6, 7). However, whether the short-term efficacy benefit of induction chemoimmunotherapy translates into a survival benefit in patients with stage III NSCLC receiving cCRT has not yet been confirmed. Before the era of immunotherapy, induction chemotherapy was confirmed to have no additional survival benefit in patients receiving cCRT (6, 7). The advantage of the downstaging effect of induction chemotherapy does not translate into a significant PFS or OS benefit. In this study, adding immunotherapy to induction chemotherapy significantly prolonged survival in patients receiving cCRT. Moreover, although calculated from the start of treatment, the 83.7% 2-year PFS rate and 91.7% 2-year OS rate in the present study were non-inferior to the 45.0% 2-year PFS rate and 66.3% 2-year OS rate in the PACIFIC trial, suggesting that induction chemoimmunotherapy before cCRT may achieve similar outcomes to consolidation immunotherapy with fewer cycles of immunotherapy. However, the small sample size limits the ability to draw a definitive conclusion. Prospective randomized controlled trials focusing on the comparison between these two treatment modalities are needed in the future.

The risk of treatment-related adverse events is another primary concern. Previous data have indicated that combining immunotherapy with CRT could increase the incidence of pneumonitis (17–19). In this study, there was no significant difference in the incidence of pneumonitis regardless of whether patients received induction chemoimmunotherapy before CRT. The incidence of grade 3/4 pneumonitis in patients receiving induction chemoimmunotherapy was approximately 3%, which was slightly lower than the findings of Wang et al. (9.3%) and the incidence of pneumonitis in the real-world PACIFIC regimen (4.4%-6.0%) (5, 20, 21). Only one-third of patients receiving induction chemoimmunotherapy in the present study received cCRT, which may have contributed to this result. The incidence of the remaining common TRAEs was similar between the two groups, except for a higher but still acceptable incidence of hematological toxicity in patients receiving induction chemoimmunotherapy. When cases were further restricted to those receiving induction treatment plus cCRT, the addition of immunotherapy did not significantly increase the incidence of grade 3/4 TRAEs, suggesting that the toxicity of induction chemoimmunotherapy plus CRT or even cCRT is tolerable.

This study represents a retrospective analysis of data and thus has some evident limitations. First, this is a retrospective single-institution study, which may limit the generalizability of the results. Second, this study spanned a relatively long period, and the improved treatment may lead to an over-interpretation of the results. Third, data regarding PD-L1 expression are sparse because it is not routinely tested in stage III NSCLC at our center. In addition, the heterogeneous treatment approach, including different ICI agents, may also bias the results. Although a previous study demonstrated no statistically significant differences in the safety and efficacy between various ICIs (22), future studies should use identical ICI agents and stratify by PD-L1 expression to minimize confounding. Furthermore, as previously mentioned, the moderate sample size of patients treated with induction chemoimmunotherapy and the subsequent analysis in the cCRT setting further reduced the sample size, which may also influence the results. Finally, this novel treatment modality was not compared with the PACIFIC regimen due to the low proportion of patients receiving consolidation immunotherapy, and future large-scale clinical trials are warranted to confirm whether it can achieve comparable outcomes to consolidation immunotherapy and whether the combination of induction and consolidation immunotherapy can provide further survival benefits. Despite these limitations, our analysis demonstrates the safety and efficacy of induction chemoimmunotherapy before CRT with relatively few cycles of immunotherapy and, more importantly, may provide a new option for patients who cannot or refuse to receive 1 or even 2 years of consolidation immunotherapy due to the high economic burden and so on. To the best of our knowledge, this is the first study to evaluate the prognostic role of induction chemoimmunotherapy in unresectable stage III NSCLC patients. We believe this study could provide a new direction for research or a treatment option for patients who cannot or refuse to receive consolidation immunotherapy.

## 5 Conclusions

In conclusion, induction chemoimmunotherapy is safe and may improve outcomes of CRT in patients with unresectable stage III NSCLC. Moreover, induction chemoimmunotherapy may further improve treatment response and survival outcomes compared to induction chemotherapy before cCRT.

## Data availability statement

The raw data supporting the conclusions of this article will be made available by the authors, without undue reservation.

## Ethics statement

The studies involving humans were approved by Institutional Review Board of Cancer Institute and Hospital of Tianjin Medical University. The studies were conducted in accordance with the local legislation and institutional requirements. The need for informed consent was waived owing to the retrospective nature of the study.

## Author contributions

SG: Conceptualization, Data curation, Formal analysis, Writing – original draft. SZ: Data curation, Formal analysis, Writing – original draft. KR: Data curation, Formal analysis, Writing – review & editing. XYL: Data curation, Formal analysis, Writing – review & editing. XL: Formal analysis, Funding acquisition, Investigation, Writing – review & editing. LZ: Conceptualization, Funding acquisition, Supervision, Writing – review & editing.
