# Supplementary material for: Induction chemoimmunotherapy may improve outcomes of chemoradiotherapy in patients with unresectable stage III NSCLC
Source: Front Immunol. 2023 Nov 27;14:1289207. doi: 10.3389/fimmu.2023.1289207 (PMC10711043; doi:10.3389/fimmu.2023.1289207)
Supplement: Supplementary file 1 [file Table_1.docx]

Table S1 Univariate and multivariate analysis for PFS

| Factor | Univariate | |  | Multivariate | |  |
| --- | --- | --- | --- | --- | --- | --- |
|  | HR | 95% CI | P | HR | 95% CI | P |
| Age |  |  |  |  |  |  |
| <65 | 1.000 (reference) | | |  |  |  |
| ≥65 | 0.869 | 0.650,1.162 | 0.344 |  |  |  |
| Sex |  |  |  |  |  |  |
| Male |  | | |  |  |  |
| Female | 0.982 | 0.691,1.395 | 0.918 |  |  |  |
| WHO histology |  |  |  |  |  |  |
| Squamous | 1.000 (reference) | | |  |  |  |
| Non-squamous | 0.995 | 0.743,1.333 | 0.974 |  |  |  |
| NOS | 0.818 | 0.335,1.996 | 0.658 |  |  |  |
| Stage |  |  |  |  |  |  |
| IIIA | 1.000 (reference) | | | 1.000 (reference) | | |
| IIIB | 1.422 | 1.065,1.897 | **0.017** | 1.330 | 0.992,1.782 | 0.057 |
| IIIC | 1.511 | 0.944,2.419 | 0.085 | 1.412 | 0.877,2.273 | 0.155 |
| CRT modality |  |  |  |  |  |  |
| Sequential | 1.000 (reference) | | |  |  |  |
| Concurrent | 0.812 | 0.610,1.080 | 0.152 |  |  |  |
| Radiation dose |  |  |  |  |  |  |
| <54 Gy | 1.000 (reference) | | |  |  |  |
| ≥54 Gy | 0.638 | 0.262,1.550 | 0.321 |  |  |  |
| Smoking history |  |  |  |  |  |  |
| Never smoked | 1.000 (reference) | | | 1.000 (reference) | | |
| Former/current | 1.331 | 0.922,1.922 | 0.127 | 1.215 | 0.832,1.775 | 0.313 |
| ECOG |  |  |  |  |  |  |
| 0 | 1.000 (reference) | | | 1.000 (reference) | | |
| 1 | 1.838 | 1.102,3.067 | **0.020** | 1.509 | 0.885,2.573 | 0.131 |
| 2 | 2.400 | 0.934,6.168 | 0.069 | 2.134 | 0.824,5.523 | 0.118 |
| Treatment modality |  |  |  |  |  |  |
| CRT | 1.000 (reference) | | | 1.000 (reference) | | |
| I-CRT | 0.549 | 0.349,0.863 | **0.009** | 0.562 | 0.357,0.885 | **0.013** |

Table S2 Univariate and multivariate analysis for OS

| Factor | Univariate | |  | Multivariate | |  |
| --- | --- | --- | --- | --- | --- | --- |
|  | HR | 95% CI | P | HR | 95% CI | P |
| Age |  |  |  |  |  |  |
| <65 | 1.000 (reference) | | |  |  |  |
| ≥65 | 1.003 | 0.710,1.415 | 0.988 |  |  |  |
| Sex |  |  |  |  |  |  |
| Male | 1.000 (reference) | | |  |  |  |
| Female | 0.880 | 0.573,1.353 | 0.562 |  |  |  |
| WHO histology |  |  |  |  |  |  |
| Squamous | 1.000 (reference) | | |  |  |  |
| Non-squamous | 0.826 | 0.575,1.186 | 0.300 |  |  |  |
| NOS | 0.937 | 0.345,2.548 | 0.899 |  |  |  |
| Stage |  |  |  |  |  |  |
| IIIA | 1.000 (reference) | | | 1.000 (reference) | | |
| IIIB | 1.635 | 1.162,2.300 | **0.005** | 1.519 | 1.073,2.150 | **0.018** |
| IIIC | 1.027 | 0.551,1.912 | 0.934 | 0.984 | 0.525,1.844 | 0.960 |
| CRT modality |  |  |  |  |  |  |
| Sequential | 1.000 (reference) | | | 1.000 (reference) | | |
| Concurrent | 1.290 | 0.929,1.793 | 0.128 | 1.254 | 0.899,1.749 | 0.183 |
| Radiation dose |  |  |  |  |  |  |
| <54 Gy | 1.000 (reference) | | |  |  |  |
| ≥54 Gy | 0.547 | 0.202,1.481 | 0.236 |  |  |  |
| Smoking history |  |  |  |  |  |  |
| Never smoked | 1.000 (reference) | | | 1.000 (reference) | | |
| Former/current | 1.466 | 0.924,2.326 | 0.104 | 1.347 | 0.836,2.169 | 0.221 |
| ECOG |  |  |  |  |  |  |
| 0 | 1.000 (reference) | | | 1.000 (reference) | | |
| 1 | 1.850 | 0.972,3.520 | 0.061 | 1.463 | 0.745,2.872 | 0.269 |
| 2 | 2.609 | 0.814,8.361 | 0.107 | 2.259 | 0.694,7.354 | 0.176 |
| Treatment modality |  |  |  |  |  |  |
| CRT | 1.000 (reference) | | | 1.000 (reference) | | |
| I-CRT | 0.472 | 0.230,0.968 | **0.040** | 0.517 | 0.251,1.066 | **0.074** |
